# Resolution of a Chiral β‐Aminoketone via Diastereomeric Salt Formation: From Experimental Evidence to Molecular‐Level Insights Into Solution‐Phase Clusters

**DOI:** 10.1002/chir.70087

**Published:** 2026-03-04

**Authors:** Caterina Momoli, Laura Palombi, Isabella Daidone, Erica Scarel, Massimiliano Aschi

**Affiliations:** ^1^ Dipartimento di Scienze Fisiche e Chimiche Università dell'Aquila L'Aquila Italy; ^2^ Dept. of Chemical and Pharmaceutical Sciences University of Trieste Trieste Italy

**Keywords:** chiral resolution, cluster size distribution, molecular dynamics simulations, prenucleation

## Abstract

The classical diastereomeric salt resolution approach was employed to separate (±)‐1,3‐diphenyl‐3‐(phenylamino)propan‐1‐one using both enantiomers of 10‐camphorsulfonic acid (CSA) as resolving agents. Gentle stirring at room temperature resulted in the stereoselective precipitation of a single diastereomeric salt, yielding a solid phase highly enriched in one enantiomer of the target compound. Control experiments confirmed the crucial role of the chiral counterion in directing the selectivity of the process. Molecular Dynamics simulations and subsequent Principal Component Analysis revealed slight but significant differences in the pre‐nucleation size distribution of ionic clusters and in the dynamics of their mutual interconversion, hence suggesting that these differences could play a role in the racemic resolution.

## Graphical abstract

Subtle but significant differences between the heterochiral and homochiral salts during the prenucleation stage, revealed by molecular simulations, are related to the enantiomeric separation of a β‐aminoketone through the precipitation of its corresponding diastereomeric salts.

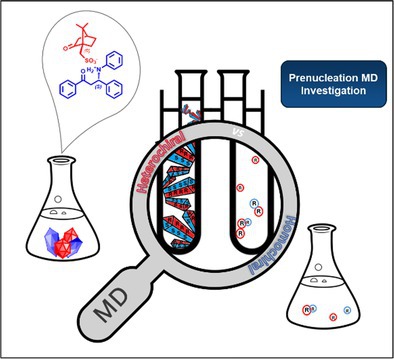

## 1 Introduction

The preparation of chiral molecules as single enantiomers remains a central challenge in organic and pharmaceutical chemistry, due to the distinct biological and physicochemical properties of nonsuperimposable mirror images [1]. Despite major advances in asymmetric synthesis in recent years [2], optical purity is most commonly achieved through enantiomer separation techniques, valued for their simplicity, cost‐effectiveness, and reliability [3, 4]. In particular, diastereomeric salt or cocrystal formation with chiral agents [5, 6], followed by fractional crystallization, constitutes a robust, general, and scalable strategy for resolving diverse chiral compounds. This approach can also complement asymmetric synthesis when stereoselectivity is suboptimal [7, 8, 9]. Research in this field remains highly active, with recent advances demonstrating that a chiral agent can simultaneously drive preferential cocrystal formation and facilitate racemization in solution [10], linking classical resolution methods with rare but conceptually appealing phenomena such as spontaneous enantioselective crystallization and Viedma ripening [11, 12, 13, 14, 15, 16].

**SCHEME 1:**
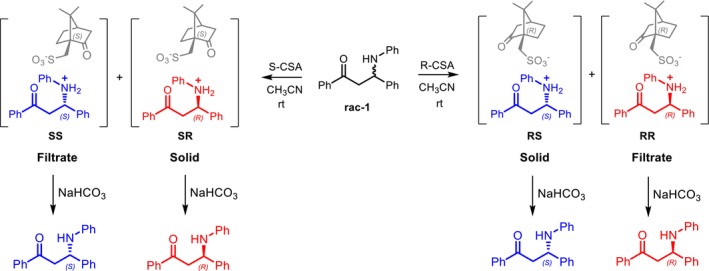
Resolution of rac‐1 using R‐CSA and S‐CSA.

In this work, we identified suitable conditions—solvent, chiral agent, molar ratios, and concentration—for the resolution of (±)‐1,3‐diphenyl‐3‐(phenylamino)propan‐1‐one (1) via diastereomeric salt formation using the low‐cost, readily available enantiomers of 10‐camphorsulfonic acid (R‐CSA and S‐CSA) as resolving agents (Scheme 1). Compound **1** was chosen as a representative of β‐aminoketones, a class of compounds of broad interest in stereoselective synthesis and medicinal chemistry. Indeed, significant efforts have been devoted over time to the asymmetric synthesis of these scaffolds, including organocatalytic approaches based on enantioselective Michael additions of anilines to chalcones [17, 18]. However, for the compound under investigation, such approaches achieved optimal enantioselectivity only for one enantiomer, as the corresponding pseudo‐enantiomeric organocatalyst failed to provide comparable results for the opposite configuration [17]. We therefore envisaged overcoming this limitation by exploiting the arylamino group of **1** to form diastereomeric salts with R‐CSA and S‐CSA, enabling access to both enantiomers in enantioenriched form. In parallel, atomistic molecular dynamics (MD) simulations were employed to gain insight into the molecular‐level origins of the observed resolution, with particular emphasis on the role of pre‐nucleation clustering. In recent years, computational approaches based on atomistic simulations have become an increasingly active area of research for addressing nucleation and crystallization processes [19, 20]. Despite significant methodological advances [21, 22, 23, 24, 25], these phenomena remain among the most challenging problems in theoretical and computational chemistry [26]. One of the main difficulties arises from the limited accuracy of semiclassical force fields in describing the weak noncovalent interactions that dominate the earliest stages of nucleation [27, 28]. Moreover, nucleation and crystallization are inherently complex, mesoscopic, and far‐from‐equilibrium processes, requiring system sizes and timescales that largely exceed the capabilities of current MD simulations [25, 29, 30, 31, 32, 33].

For these reasons, we focused our attention on pre‐nucleation clustering under equilibrium conditions. To this end, we examined the distribution of nanoclusters formed by compound **1** with enantiopure R‐CSA and S‐CSA in acetonitrile solution, just below saturation. In line with recent studies [33, 34, 35, 36, 37, 38, 39, 40, 41, 42, 43, 44, 45], this approach provides a valuable perspective on pre‐nucleation processes and their potential role as the incipient stage of the complex crystallization phenomenon.

## 2 Results and Discussion

**FIGURE 1:**
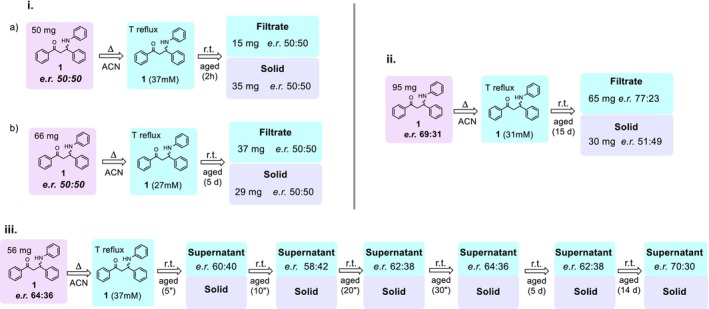
Monitoring **1'**s enantiomeric ratio (e.r.) of racemic (i.) and scalemic samples (ii. and iii.) at defined time points in ACN‐saturated solutions.

We first examined the feasibility of direct deracemization of compound **1** by investigating its crystallization behavior in both racemic and scalemic forms. In a typical experiment, **1** was dissolved in ACN under stirring after brief heating, then slowly cooled to room temperature until precipitation occurred. When **rac‐1** was used, chiral HPLC analysis of both the solid and the filtrate showed no change in enantiomeric excess, indicating no kinetic or thermodynamic preference toward either enantiomer (Figure 1 i). When a scalemic sample was employed, minimal fluctuations in the enantiomeric ratio of the supernatant were detected over time (Figure 1 ii–iii). These changes, falling within experimental uncertainty, are consistent with a dynamic but neither systematic nor directional solid–solution exchange. Upon prolonged standing (14–15 days), the solution may become slightly enriched in the predominant enantiomer (ca. 6%), a trend consistently observed in both ii and iii experiments of Figure 1. Although further experiments would be required to quantitatively confirm any preferential precipitation of **1** as a racemate, these data suggest that any such effect would be marginal and manifest only over extended timescales.

Treatment of rac‐1 with enantiopure CSA in CH_3_CN leads to selective formation of diastereomeric ammonium camphorsulfonate salts with partial precipitation. Cleavage of the isolated salts with NaHCO_3_ affords the corresponding enantiomers of 1.

We next investigated the resolution of compound **1** as ammonium salt, using both chiral and achiral acids (Table 1). Given the well‐established utility of 10‐camphorsulfonic acid in amine resolution, both enantiomers were evaluated as chiral resolving agents.

**TABLE 1:** Attempts to resolve rac‐**1** as ammonium salt under different conditions^a^.

Entry	rac‐1 mg/[C]	Acid (eq.)	Filtrate		Solid	
			mg	e.r.	mg	e.r.
1	100/22 mM	*R‐CSA* (0.5)	30	32:68	70	57:43
2	100/22 mM	*R‐CSA* (1.1)	69	36:64	29	95:5
3	60/28 mM	*R‐CSA* (1.1)	40	30:70	19	93:7
4	104/66 mM	*R‐CSA* (1.1)	27	44:56	65	54:46
5^b^	60/28 mM	*S‐CSA* (1.1)	40	68:32	18	10:90
6^c^	18/28 mM	*S‐CSA* (1.0)	Not recovered		14	2:98
7	100/22 mM	HCl (1.2)	50	50:50	45	50:50
8	50/66 mM	PhCO_2_H (1.0)	19	50:50	30	50:50
9^d^	50/28 mM	PhCO_2_H (1.1)	38	54:46	13	52:48
10	60/25 mM	p‐TsOH (1.1)	44	51:49	15	52:48

^a^ Rac‐**1** was treated with the indicated acids in CH_3_CN under the different conditions. After salt formation, the mother liquor and the precipitate were separated, each converted back to the free base by treatment with NaHCO_3_. **1** was then recovered by extraction with CH_2_Cl_2_, and the enantiomeric ratio (e.r.) in both fractions (filtrate and solid, respectively) were determined by chiral HPLC.

^b^ Starting **1** e.r. 48:52.

^c^ Starting **1** e.r. = 10:90.

^d^ Starting **1** e.r. 52:48.

Addition of R‐CSA to a suspension of **1** in acetonitrile rapidly afforded a clear solution, indicating proton transfer and salt formation, followed by precipitation. The precipitate and mother liquor were separated by filtration, and the corresponding free bases were regenerated by treatment with aqueous NaHCO_3_ and extraction with CH_2_Cl_2_ (see the Supporting Information for details). Chiral HPLC analysis showed that the solid phase was enriched in one enantiomer of **1**, while the filtrate was enriched in the opposite enantiomer (entries 2–3). Conversely, treatment with S‐CSA afforded an inverted selectivity (entries 5–6), mirroring the behavior observed with R‐CSA.

By normalizing the absolute amounts (mg) of enriched enantiomers obtained in the individual experiments to the theoretical maximum for a single enantiomer and correcting for enantiomeric purity, entry 3 was found to provide the best compromise between mass recovery and enantiomeric purity. More diluted conditions (entry 2) resulted in a slight increase in enantiomeric ratio at the expense of mass recovery, while lower acid loading (entry 1) or more concentrated conditions (entry 4) led to largely nonselective precipitation. Additional details on the calculation of mass recoveries are provided in the Supporting Information. To further confirm that the observed enantioselectivity originates from the chiral counterion, control experiments were carried out using achiral acids under similar conditions (Table 1, entries 7–10). In all cases, the initial suspension rapidly turned into a clear solution upon acid addition, consistent with salt formation; however, within experimental error, both the crystallized solid and the filtrate retained the enantiomeric ratio of the starting material, underscoring the crucial role of the chiral acid.

**FIGURE 2:**
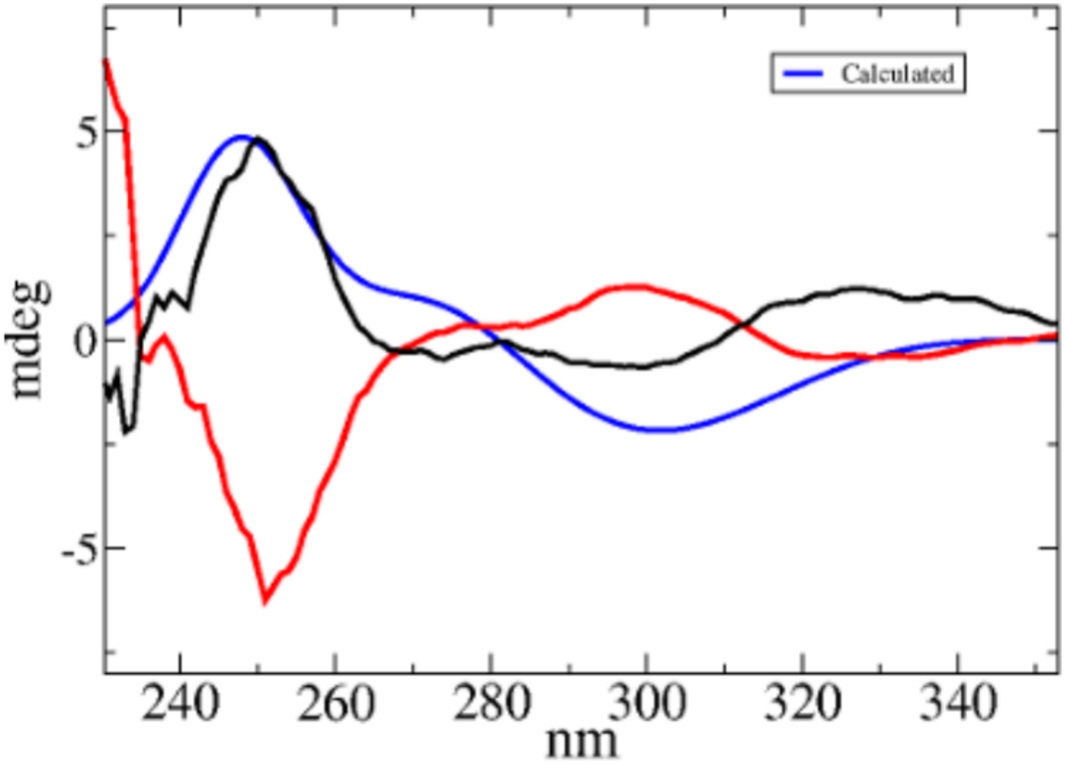
Red and black lines: experimental ECD spectra of **1** obtained from the solid of entries **2** (e.r. 95:5) and **6** (e.r. 2:98), respectively. Blue line: calculated spectrum of *(R)*‐**1**. Additional details on the experimental and calculated spectra can be found in Section S4.

Because no crystallographic data or other absolute configuration assignments are available for either the enantiomers of **1**, the absolute configurations of the enantiomers obtained from the R‐CSA and S‐CSA resolution experiments were determined by comparing their experimental electronic circular dichroism (ECD) spectra with the calculated spectrum of *(R)*‐1 (Figure 2) whose details can be found in Tables S3–S4 and Figures S1–S4.

As shown in Figure 2, the calculated ECD spectrum of (R)‐1 is in good agreement with the experimental ECD spectrum of the enantiomer isolated from the solid of entry 6 (Table 1), displaying the same sign pattern of the main cotton effects. These results indicate that chiral resolution with R‐CSA leads to the preferential precipitation of the heterochiral salt containing the (S)‐1 enantiomer, whereas chiral resolution with S‐CSA favors precipitation of the (R)‐1 enantiomer. Once the absolute configurations of **1** were unambiguously assigned, MD simulations of the corresponding diastereomeric salts in solution were performed to gain some insight into the molecular origin of the observed differences in solubility in acetonitrile. Each simulation contained the same number of molecular ions (22 cations and 22 anions), representing all possible ion‐pair combinations between **rac‐1** and R‐CSA or S‐CSA in acetonitrile (Figure 1).

Specifically, (R)‐1 was simulated separately with R‐CSA and S‐CSA, and likewise (S)‐1 with R‐CSA and S‐CSA, resulting in four independent systems hereafter denoted as RR, RS, SR, and SS, respectively. This comprehensive setup was chosen to allow a consistent comparison among all plausible diastereomeric combinations formed upon treatment of rac‐1 with R‐CSA or S‐CSA in acetonitrile. The four MD simulations were subsequently analyzed to identify possible differences in cluster populations and dynamical behavior indicative of distinct prenucleation conditions. To this end, we specifically developed an analysis approach, which is presented in the “Materials and Methods” section.

**FIGURE 3:**
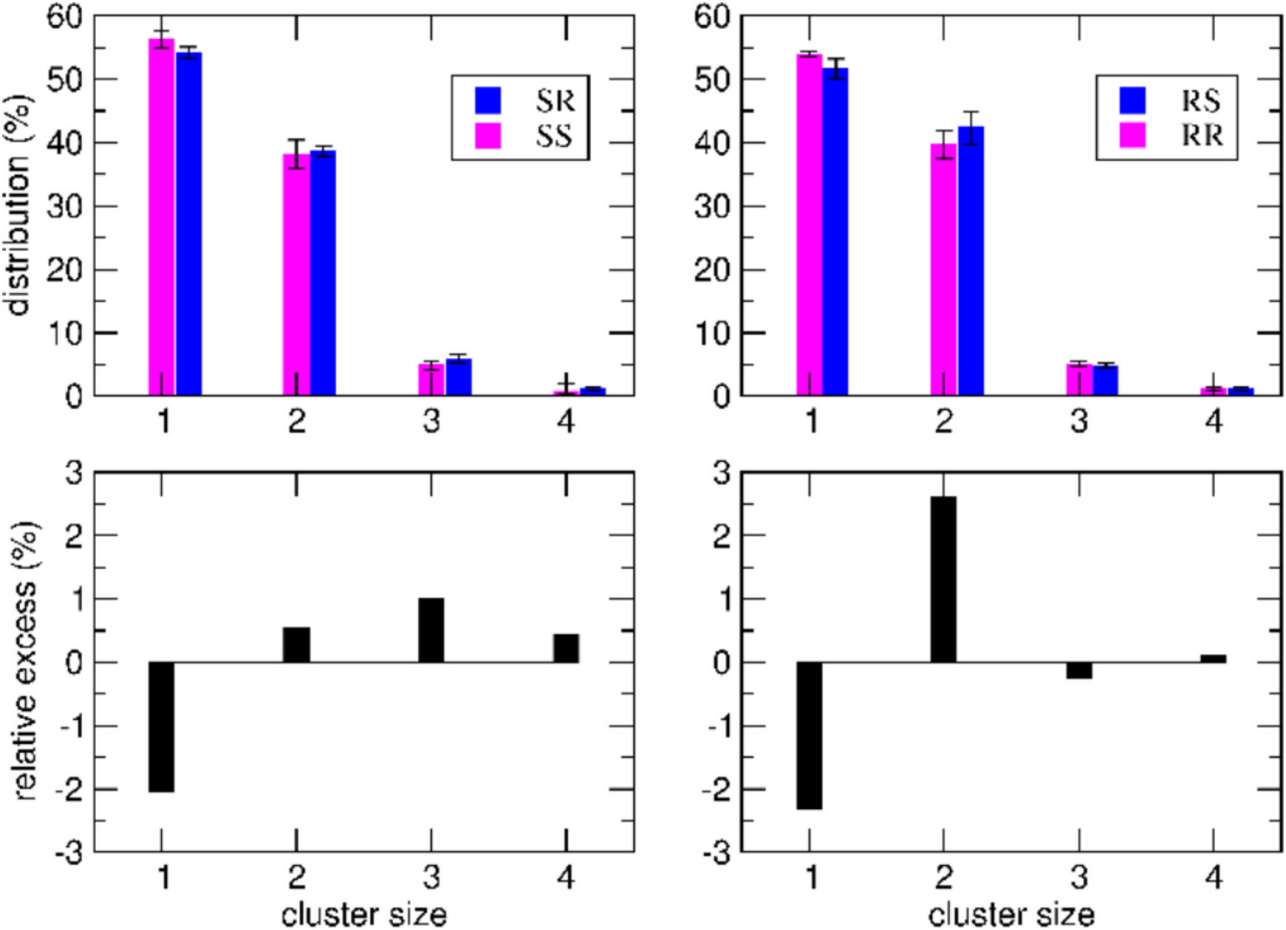
Top panels: Cluster size distributions monitored over the MD simulations. Bottom panels: Population differences between SR/SS (left) and RS/RR (right) systems. See Table S1 for additional details.

The results on the formation of ionic clusters, along with the corresponding size population analysis, are summarized in Figure 3.

The complete set of results is provided in Table S1. In the top panels, we report the cluster‐size distributions computed along the respective MD simulations. All the data are reported with the corresponding uncertainty evaluated as a maximum error. Across all four simulated systems, approximately half of the ions remain free in solution (cluster size = 1). The remaining ions form clusters, with dimers and trimers being the most prevalent, followed by smaller populations of tetramers. Larger clusters are negligibly populated and are therefore not shown in the figure. Notably, this distribution—where approximately 50% of the ions remain unpaired, indicating a limited tendency to form cluster—is consistent with the experimental observations, as at the working concentrations, most of the material remains dissolved (Table 1). From the figure, it is evident that the differences in monomer populations, that is, the probability of finding free ions at equilibrium, although small, as expected from the experimental outcomes, are statistically significant. The bottom panels of Figure 3 display the differences in cluster populations between each pair of diastereoisomers. As shown, the variations in monomer populations, that is, the probability of finding free ions at equilibrium, are statistically significant. Specifically, the difference between the average number of free monomers is 1.3 ± 0.9 for the RS/RR pair and 1.0 ± 0.7 for the SR/SS pair. Moreover, our results indicate that, for the systems obtained with either the R‐ or *S‐CSA*, the heterochiral salts, namely, RS and SR, also show a greater tendency to form clusters, particularly dimers and trimers. Moreover, as illustrated in Figure 3, the differences observed within each pair, although subtle, are statistically significant within the estimated uncertainties, which—as noted above—were evaluated as maximum errors. These findings highlight measurable variations in clustering behavior and further support the overall consistency and reliability of our results.

**FIGURE 4:**
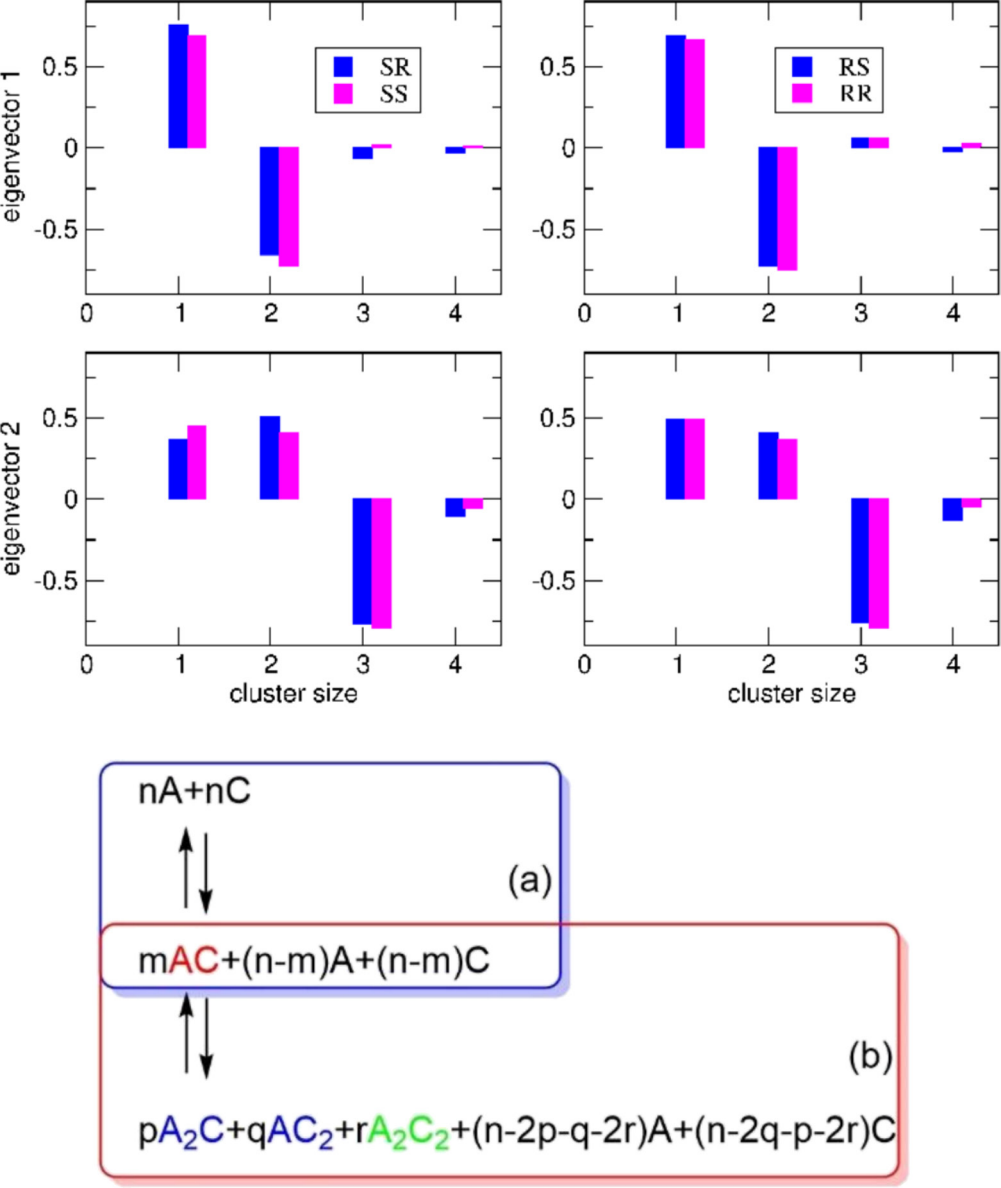
Upper panel: Composition of the first two eigenvectors obtained from PCA of the cluster population distributions in the four MD simulations. For each diastereomeric pair (SR, RS, SS, and RR), the contributions of free ions (cluster size 1) and of clusters with sizes 2–4 (dimers, trimers, and tetramers) are reported. Clusters larger than size 4 were neglected, as their contributions were negligible in all relevant eigenvectors. Lower panel: Schematic representation of the dominant fluctuation modes described by the first and second eigenvectors obtained from the PCA, corresponding to those reported in upper panels.

To further explore the mechanisms underlying the formation of clusters, we investigated the clustering dynamics of the four systems under prenucleation conditions. To this end, we performed a principal component analysis (PCA) of the cluster populations, as described in the “Materials and Methods” section. The first two eigenvectors, which account for approximately 88%–91% of the total variance and thus capture the essential features of the clustering dynamical equilibrium, are shown in Figure 4 (the complete set of results is reported in Table S2).

From the analysis of the components of the first eigenvector (top‐left column of Figure 4), an anticorrelation emerges between monomers and dimers indicating the dominant fluctuation between dissociation and ion pairing (see scheme (a) in panel B of Figure 4).

The analysis of the components of the second eigenvector (right column of Figure 4) shows an anticorrelation between monomers–dimers and trimers–tetramers, hence indicating the possible emergence of larger clusters from smaller ones (see scheme (b) in panel B of Figure 4).

The first eigenvector contrasts free ions and dimers, reflecting the dominant fluctuation between dissociation and ion pairing (scheme (a) Figure 4). The second eigenvector shows an anticorrelation between monomers–dimers and trimers–tetramers (scheme (b) Figure 4). This partition reflects a physically meaningful distinction between small, highly dynamic species dominated by ion pairing (monomers and dimers) and larger, more structured ionic clusters (trimers and tetramers). The observed anticorrelation therefore represents a redistribution of population from smaller to larger clusters.

Consistent with the experimentally observed solubility trends, the less soluble heterochiral RS and SR pairs exhibit a higher contribution of tetramers to the second eigenvector, approximately twice that observed for the RR and SS pairs. This difference indicates a subtle but significant enhancement in the dynamical tendency to form larger clusters in the heterochiral systems.

It should be noted that MD simulations inherently suffer from several limitations, including force‐field inaccuracies and incomplete sampling, which introduce systematic errors. However, their impact can be mitigated by focusing on relative differences between simulations rather than on absolute values. In this context, the observed population shifts between heterochiral and homochiral salts, although small in absolute terms, are consistent across both pairs (RS/RR and SR/SS), supporting their statistical significance and mechanistic relevance.

## 3 Conclusions

This study reports, for the first time, the successful resolution of (±)‐1,3‐diphenyl‐3‐(phenylamino)propan‐1‐one using (R)‐ and (S)‐camphorsulfonic acid as resolving agents. The absolute configuration of the resulting enantiomers has also been determined for the first time by ECD analysis.

To explore possible molecular‐level differences among the systems investigated, we performed MD simulations to characterize the equilibrium populations of clusters of different sizes in solution under prenucleation conditions. For this purpose, we developed an MD‐based protocol capable of identifying transient clusters along the simulations and, consequently, determining their size distribution and interconversion dynamics.

Consistent with the preferential precipitation of the heterochiral salts observed experimentally, the simulations reveal subtle yet significant differences already in the pre‐aggregation regime. In particular, the heterochiral species, that is, the less soluble salts, exhibited a slightly higher tendency to form clusters—especially trimers and tetramers. These findings suggest that even minor variations in solution‐phase behavior may influence the macroscopic outcome of chiral resolution.

## 4 Materials and Methods

Details of the experimental procedures are reported in Section S1. A major goal of the present work was to develop an operational criterion, to be used in conjunction with MD simulations, to identify the formation and evolution of clusters, while acknowledging the inherent arbitrariness of such definitions. This section describes the proposed method in detail.

**FIGURE 5:**
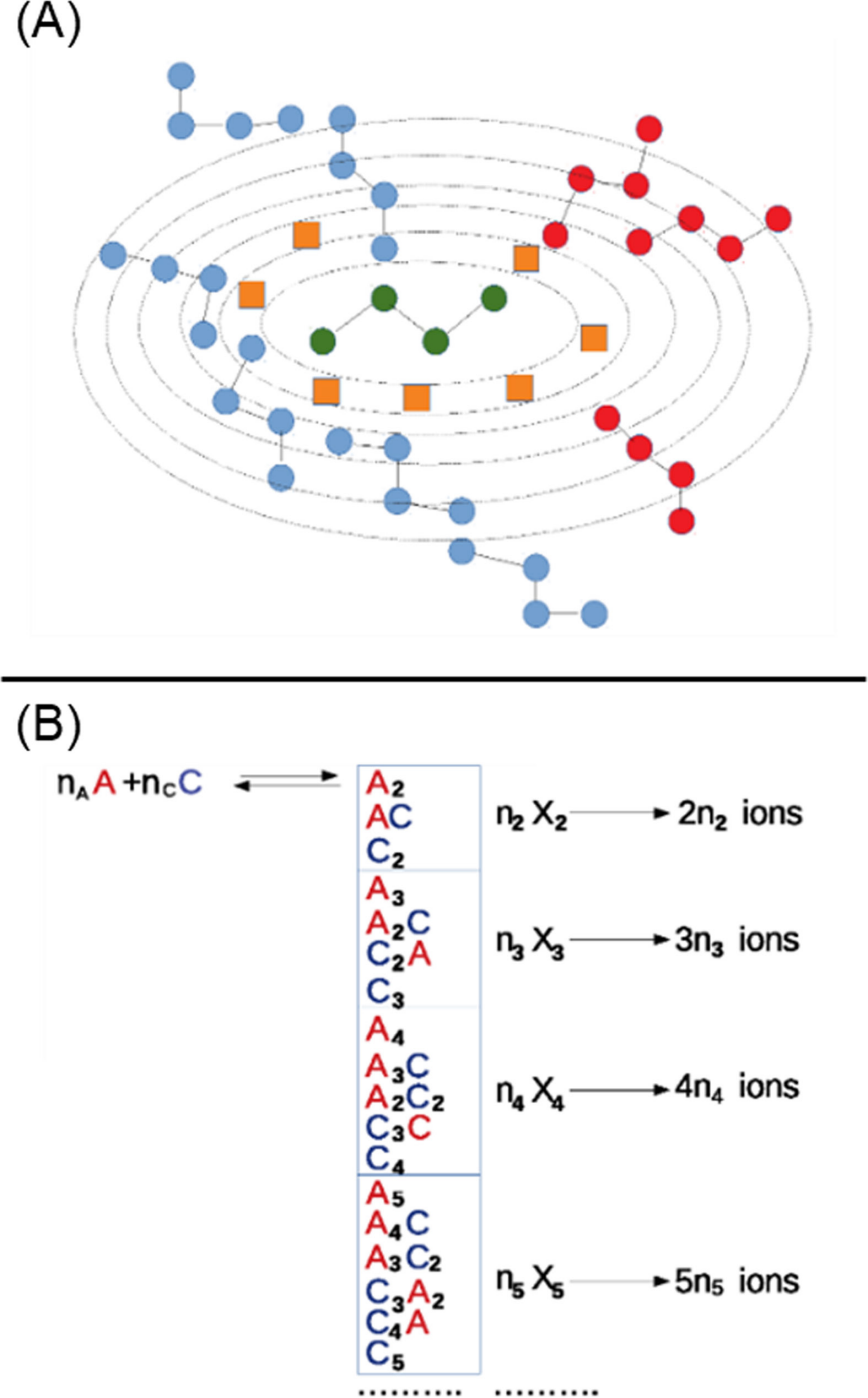
(A) Schematic representation of the method used to define the formation of instantaneous clusters. The reference ion is shown in green, ions belonging to the same cluster in blue, and ions excluded from the cluster in red. Solvent molecules are schematically shown as orange squares. (B) Schematic view of the basis set used to define the vector state in this study. For simplicity, only clusters up the ×5 are shown.

The central idea is that two particles are considered to be in contact and thus to contribute to the same cluster (of any size), if their closest approach is not obstructed by any solvent molecule or part thereof. The conceptual basis of this definition is illustrated schematically in Figure 5A.

Operatively, at each MD timestep, we proceed as follows:

- *Selection of Reference Ion and Construction of the Best‐Fitting Ellipsoid*
  One reference ion (either cation or anion) is chosen, and the best‐fitting ellipsoid is constructed by diagonalizing the 3 × 3 geometrical covariance matrix of its cartesian coordinates [46], with eigenvalues, *λ*_i_, providing the semiaxis lengths of the ellipsoid (*a*_*i*_ = 2√*λ*_*i*_).
- *Generation of Concentric Ellipsoidal Shells and Population Analysis*
*n* concentric ellipsoidal shells are constructed around the reference ion with semiaxes given by *a*(*n*) = *a*_*i*_ + *n δ*, where *δ* equal to 0.4 nm in the present study roughly corresponds to the size of one solvent molecule. Starting from the inner shell, the number of atoms belonging to ions (both cations and anions) and solvent molecules is counted. The iterative shell expansion is terminated when either (a) the outermost shell contains only atoms belonging to solvent molecules or (b) the atoms belonging to the ions found are not in contact with those in any of the inner shells. For this latter argument, we used a different metric essentially based on the condition that the minimum distance between the ion to be included or excluded and all the ions found in the inner shell does not exceed a threshold value of 0.3 nm. We also wish to note that the cluster distribution is not sensitive to the choice of the sequence of reference ions.
- *Definition of a Cluster*
  Once the counting process is complete, the cluster associated with the reference ion is fully defined by reconstructing all ions from the atoms found in all the analyzed ellipsoidal shells.
  When the cluster definition for a given reference ion is completed, the procedure is repeated from steps 1 to 3 for another ion, excluding those already assigned to a cluster. After all ions are mapped, the analysis advances to the next MD frame.
  Using the ellipsoidal‐shell approach described above, we determine for each trajectory frame the fraction, ni, of clusters of size “i” (with the corresponding cluster denoted as Xi) (see Figure 5B). From these values, the total fractions of clustered (ini) and free ions (n1 = nA + nC) can be obtained. These quantities define the state vector *v*(*n*_1_, 2*n*_2_, 3*n*_3_, 4*n*_4_, 5*n*_5_…) subject to the normalization condition *n*_1_ + 2*n*_2_ + 3*n*_3_ + 4*n*_4_ + 5*n*_5_ + … = 1. For simplicity, clusters of identical size but different net charges (e.g., A_2_, AC, and C_2_) are grouped together as members of the same cluster class (in the example, the cluster class *X*_2_). This approximation does not significantly influence the results (see Table S1). By aggregating data over all trajectory frames, we can construct the statistical distribution of these vector states and compare them across different MD simulations. In the present work, we derive from the procedure described above the cumulative information on the average number of ions identified as free species or as components of clusters of different sizes.
- *Dynamical Analysis via PCA*
  To gain insight into the temporal evolution and collective behavior of cluster formation, for each MD simulation, we applied PCA to the state vectors *v*(*n*_1_, 2*n*_2_, 3*n*_3_, 4*n*_4_) across all trajectory frames. The covariance matrix C was constructed according to the standard equation:
$$C_{i,j}=\frac{1}{N_{\text{frames}}}\sum_{k=1}^{N_{\text{frames}}}\left({\text{in}}_{i,k}-\hat{{\text{in}}_{i}}\right)\left({\mathrm{jn}}_{j,k}-\hat{{\mathrm{jn}}_{j}}\right)$$
  Diagonalization of C yields the eigenvalue spectrum and corresponding eigenvectors, which describe the principal modes of fluctuation in cluster populations. The eigenvalue distribution and eigenvector compositions, along with a brief discussion, are provided in the Supporting Information.

## 5 Computational Details

All the simulations were performed using the GROMACS program [47] in the NVT ensemble. The velocity‐rescaling algorithm [48] was adopted to maintain the temperature constant at 298 K, and the size of the box was adjusted to ensure the correct density of the system at the pressure of 1.0 atm. All the bonds were constrained with the LINCS algorithm [49], and the particle mesh Ewald method [50] with 34 wave vectors in each dimension and a fourth order cubic interpolation was used for long‐range electrostatics. The topologies of all the simulated species were taken from the Automated Topology Builder repository [51]. Additional details are reported in the Supporting Information.

## Author Contributions

**Caterina Momoli:** investigation. **Laura Palombi:** conceptualization, writing – original draft, writing – review and editing. **Isabella Daidone:** formal analysis, writing – review and editing, software. **Erica Scarel:** investigation. **Massimiliano Aschi:** conceptualization, writing – original draft, software, writing – review and editing, formal analysis.

## Supporting information




**Data S1:** Supporting Information.

## Data Availability

The data that support the findings of this study are available on request from the corresponding author. The data are not publicly available due to privacy or ethical restrictions.
