# Supplementary material for: Resolution of a Chiral β‐Aminoketone via Diastereomeric Salt Formation: From Experimental Evidence to Molecular‐Level Insights Into Solution‐Phase Clusters
Source: Chirality. 2026 Mar 4;38(3):e70087. doi: 10.1002/chir.70087 (PMC12960070; doi:10.1002/chir.70087)
Supplement: Supplementary file 1 — Data S1: Supporting Information. [file CHIR-38-e70087-s001.pdf]

SUPPLEMENTARY INFORMATION for

Resolution of a chiral β-aminoketone via diastereomeric salt formation: from experimental evidence to molecular-level insights into solution-phase clusters

Index.

S1. Experimental details

S2. Supplementary results from the analysis of the cluster size distribution

S3. Principal Component Analysis.

S4. ECD spectra: details and computational modelling.

S1. Experimental details.

- *General.*

The reactions were monitored by thin-layer chromatography (TLC) using Merck silica gel 60 F254 plates, and the visualization of the spots was carried out under fluorescence with a UV lamp at 254 nm. HPLC analyses were performed on a Waters 1525 HPLC system using a Chiral Art Amylose-C Neo column at λ = 254 nm.

NMR spectra were recorded on a Bruker Avance 600 spectrometer (600 MHz for ^{1}H; 101 MHz for ^{13}C), referencing the $CDCl_3$ peaks (7.26 ppm for ^{1}H; 77.00 ppm for ^{13}C).

- *Synthesis of 1,3-diphenyl-3-(phenylamino)propan-1-one; rac 1*

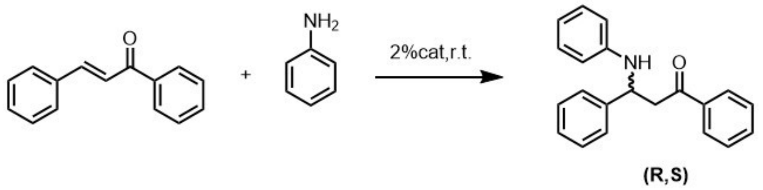

Rac-1 was synthesized following the procedure reported by Scettri et al[i]

In a 4 mL vial, 105 mg of 1,3-diphenylprop-2-en-1-one (0.5 mmol, 1 equiv.) were reacted with 91 μL of aniline (1.0 mmol, 2 equiv.) and 3 mg of Hydroquinine catalyst (0.01 mmol, 0.02 equiv.). The mixture was stirred at room temperature for 24 hours. The reaction was monitored by thin-layer chromatography eluted with a hexane: ethyl acetate 8:2 mixture. The product was obtained quantitatively, pure and racemic, after crystallization from hot ethyl acetate and hexane.

- *Typical procedure for the resolution of rac-1with R-CSA (Table 1 of the main text, entry 3)*

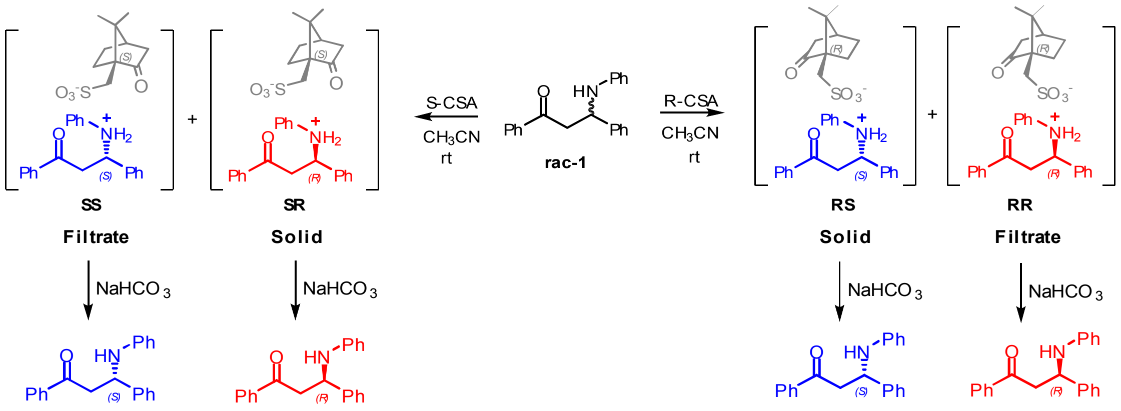

In a 10 mL vial, 60 mg (0.20 mmol) of rac- 1,3-diphenyl-3-(phenylamino)propan-1-one were suspended in 7 mL of acetonitrile. To this suspension, 51 mg (0.22 mmol, 1.1 equiv.) of (R)-CSA

were added, and the mixture was stirred at room temperature overnight. The resulting solid was collected by filtration, and the mother liquor was concentrated to dryness under reduced pressure. Both the isolated solid and the residue from the mother liquor were separately treated with 3 mL of saturated aqueous $NaHCO_3$ and extracted twice with 5 mL portions of CH_2Cl_2 to regenerate the free amine. The combined organic layers were dried over anhydrous Na_2SO_4, filtered, and concentrated under reduced pressure to afford the corresponding enantiomer-enriched fractions.

Solid fraction (from the precipitate): 19 mg, e.r. 93:7

Filtrate fraction (from the mother liquor): 40 mg, e.r. 30:70

For the preparation of the **R** enantiomer, the same procedure described above was used, employing *S*-CSA (51 mg) and **1** (60 mg e.r. 48:52) (Table 1, entry 5).

Solid fraction (from the precipitate): 18 mg, e.r. 10:90

Filtrate fraction (from the mother liquor): 40 mg, e.r. 68:32

- *NMR Characterization*

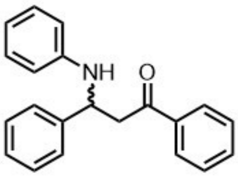

(R,S)

H NMR (600 MHz, $CDCl_3$) δ 7.97-7.91 (m, 2H), 7.62-7.55 (m, 1H), 7.50-7.44 (m, 4H), 7.38-7.33 (m, 2H), 7.29-7.24 (m, 1H), 7.15-7.10 (m, 2H), 6.74-6.70 (m, 1H), 6.63 (m, 2H), 5.05 (t, *J* = 6.3 Hz, 1H), 3.68 – 3.40 (m, 2H).

^{13}C NMR (151 MHz, $CDCl_3$) δ 198.2, 146.6, 142.70, 136.7, 133.5, 129.2, 128.9, 128.7, 128.2, 127.5, 126.5, 118.2, 114.2, 55.2, 46.2.

HPLC was performed using a Chiral Art Amylose-C Neo column, eluted with a hexane:isopropanol 95:5 mixture at a flow rate of 1 mL/min. The *S* enantiomer eluted at R_t = 19.87 min**,** and the *R* enantiomer eluted at R_t = 22.05 min**.**

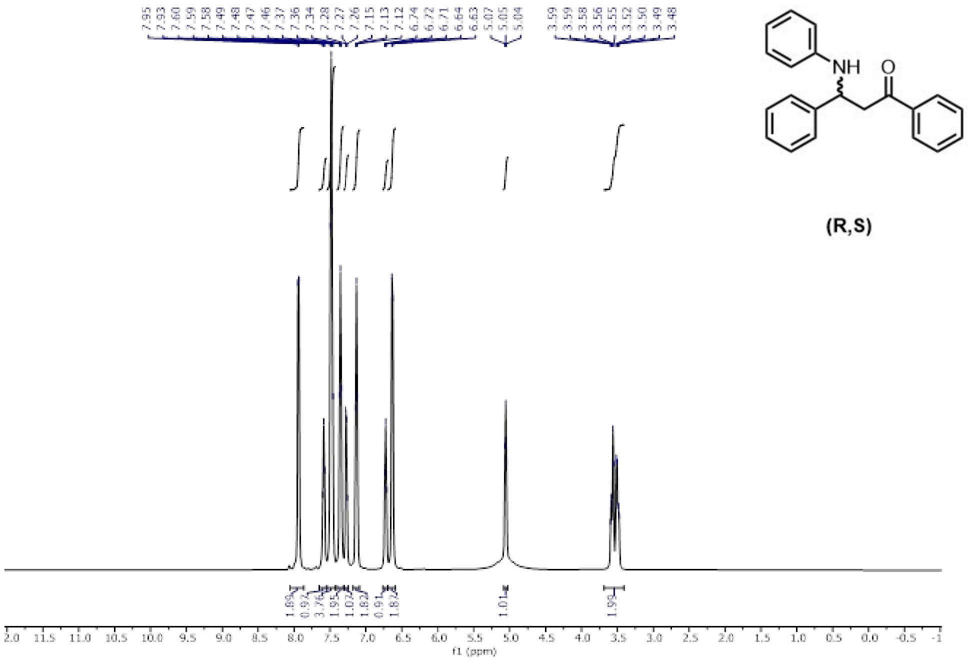
NH
O
(R,S)
f1 (ppm)

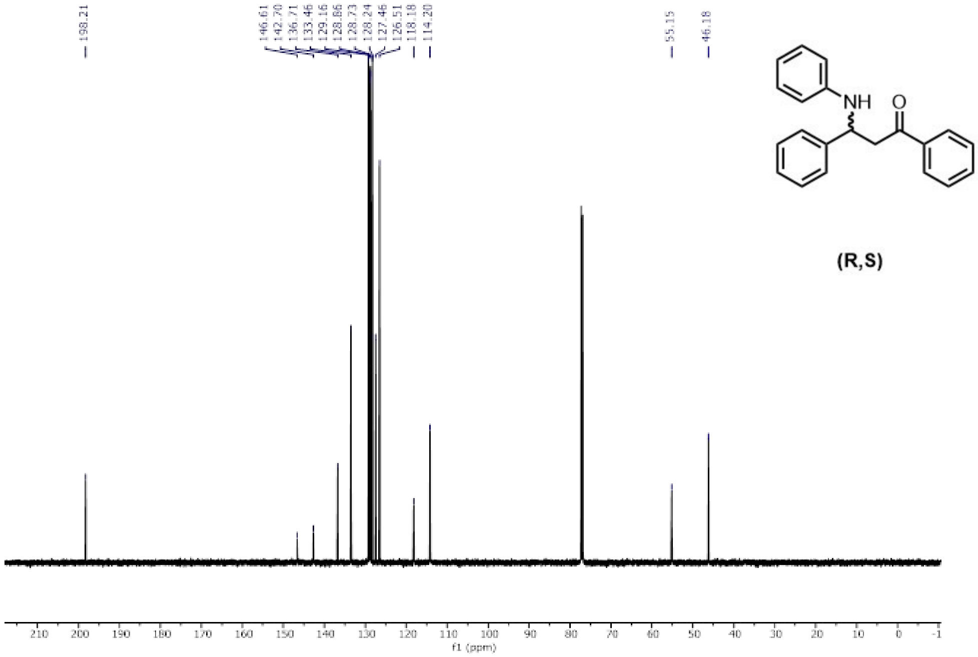
NH
O
(R,S)
f1 (ppm)

- HPLC Characterization

HPLC was performed using a Chiral Art Amylose-C Neo column, eluted with a hexane:isopropanol 95:5 mixture at a flow rate of 1 mL/min. The *S* enantiomer eluted at R_t = 19.87 min**,** and the *R* enantiomer eluted at R_t = 22.05 min**.**

Rac1

Univaq

Project Name: AmiloseCNeoChiralArt

Reported by User: System

SAMPLE INFORMATION

Sample Name:	cm16 tris-racemo	Acquired By:	System
Sample Type:	Unknown	Sample Set Name:	
Vial:	1	Acq. Method:	95 5 1ml
Injection Volume:	10,00 ul	Run Time:	45,00 Minutes

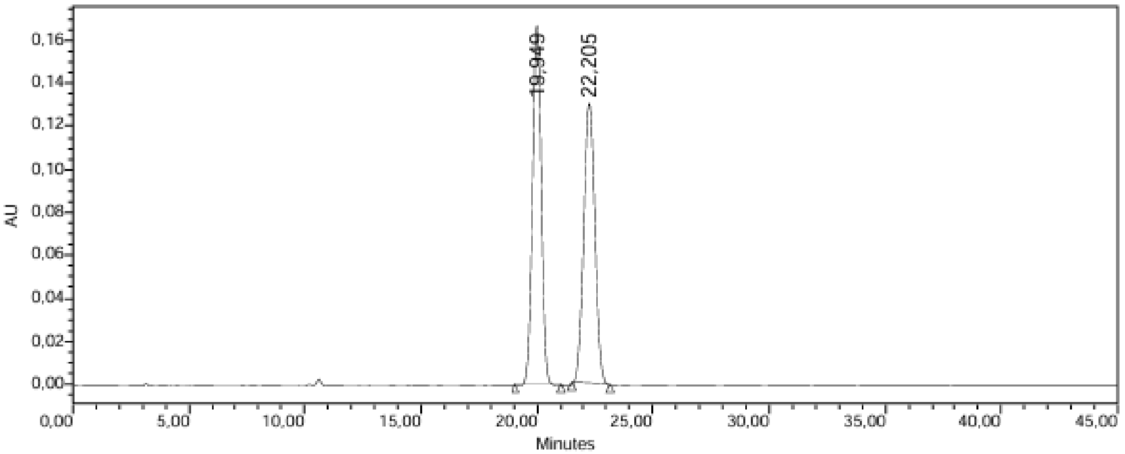

Channel: 2487Channel 1 Channel Description: Injection: 1 Date Acquired: 05/09/2025 10.13.30

Processing Method: 95 5 1ml UV Ch1

	Injection	RT (min)	Area (&V*sec)	% Area	Height (&V)	Amount	Units
1	1	22,205	4380000	49,93	130634		
2	1	19,949	4392392	50,07	167517		
Mean		21,077	4386195,685		149075,469		
% RSD		7,568	0,20		17,50		
Std. Dev.		1,60	8762,376		26080,876		

S-Enantiomer

Univaq
Project Name: AmiloseCNeoChiralArt
Reported by User: System

SAMPLE INFORMATION

Sample Name:	R-CSA-98:2	Acquired By:	System
Sample Type:	Unknown	Sample Set Name:	
Vial:	1	Acq. Method:	95 5 1ml
Injection Volume:	10,00 ul	Run Time:	45,00 Minutes

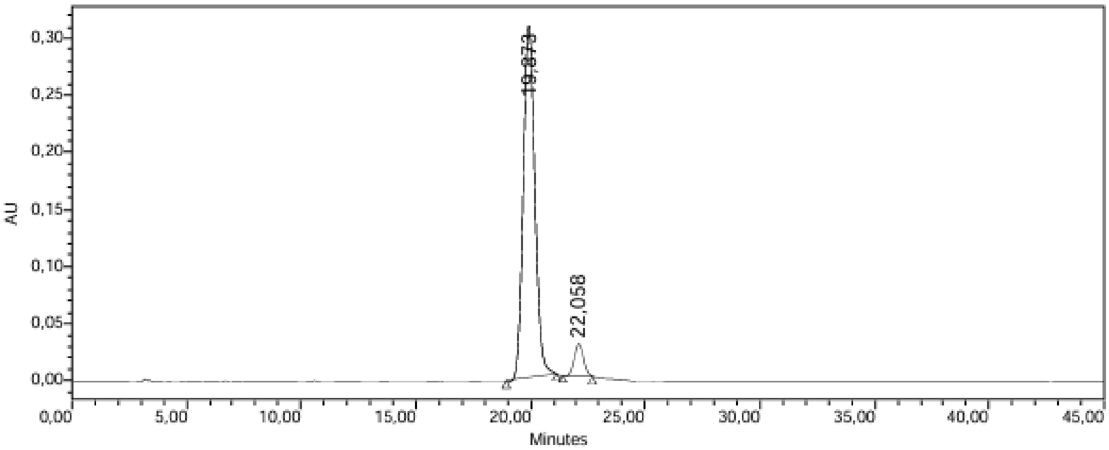

Channel: 2487Channel 1 Channel Description: Injection: 3 Date Acquired: 29/07/2025 16.05.49
Processing Method: 95 5 1ml UV Ch1

	Injection	RT (min)	Area (&V*sec)	% Area	Height (&V)	Amount	Units
1	3	19,873	10890416	93,02	309281		
2	3	22,058	817300	6,98	28538		
Mean		20,966	5853858,477		168909,371		
% RSD		7,369	121,68		117,53		
Std. Dev.		1,55	7122768,639		198515,327		

R-Enantiomer

Univaq
Project Name: AmiloseCNeoChiralArt
Reported by User: System

SAMPLE INFORMATION

Sample Name:	CM29 tetra solido 2nd crist	Acquired By:	System
Sample Type:	Unknown	Sample Set Name:	
Vial:	1	Acq. Method:	95 5 1ml
Injection Volume:	10,00 ul	Run Time:	45,00 Minutes

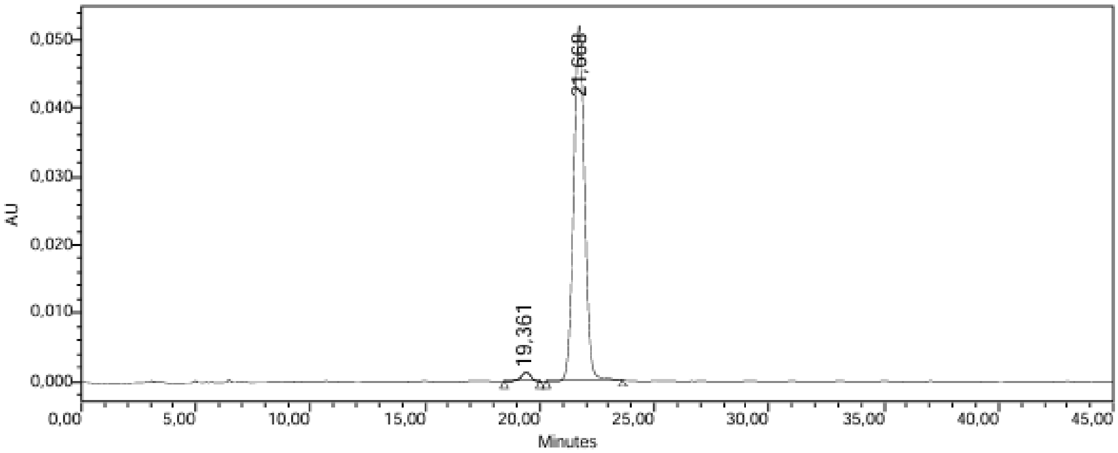

Channel: 2487Channel 1 Channel Description: Injection: 1 Date Acquired: 29/07/2025 14.28.16
Processing Method: 95 5 1ml UV Ch1

	Injection	RT (min)	Area (&V*sec)	% Area	Height (&V)	Amount
1	1	19,361	39694	2,23	1347	
2	1	21,668	1742831	97,77	52163	
Mean		20,515	891262,175		26755,018	
% RSD		7,955	135,12		134,30	
Std. Dev.		1,63	1204299,764		35932,753	

S2. Supplementary results from the analysis of the cluster size distribution

In the Table S1 below we have reported the average values with the associated maximum errors. This latter error (Δx_i) was estimated for each i-th entry of the table using the standard formula

$\Delta x_i = (X_{i,traj-2} - X_{i,traj-1})/2$

obtained upon repeating the same analysis in two halves of each trajectory (*traj-1* and *traj-2*) and calculating, for each of the them, the corresponding average value of the i-th entry (i.e. $X_{i,traj-1}$ and $X_{i,traj-1}$).

Table S1. Average number of ions found as free ions and in clusters of different sizes and charges as aobtained by the application of the method described in S1 to the four MD simulations. A and C indicate the anions and cations, respectively. Thhe error bars essentially correspond to a maximum error.

**Species**	**RR**	**RS**	**SR**	**SS**
Free ions (A + C)	23.8 ± 0.2	22.5 ± 0.9	23.9 ± 0.3	24.9 ± 0.6
A_2	0.18 ± 0.02	0.16 ± 4E-02	0.18 ± 5E-02	0.2 ± 0.1
AC	17 ± 1	18 ± 2	16.6 ± 0.4	16 ± 1
C_2	0.4 ± 0.2	0.2 ± 0.1	0.4 ± 0.2	0.37 ± 2E-02
A_3	2E-04 ± 1E-04	6E-04 ± 3E-04	n.d.	6E-04 ± 5E-04
A_2C	1.1 ± 0.1	0.9 ± 0.2	0.7 ± 0.3	0.4 ± 0.3
C_2A	1.1 ± 0.2	1.1 ± 1E-01	1.3 ± 1E-01	1.9 ± 0.3
C_3	5E-03 ± 4E-03	1E-03 ± 2E-04	2E-03 ± 1E-03	9E-04 ± 3E-04
A_3C	1.0E-02 ± 6E-03	7.5E-03 ± 4E-04	2E-02 ± 1E-02	1.1E-02 ± 9E-03
A_2C_2	0.4 ± 0.1	0.7 ± 1E-01	0.30 ± 9E-02	0.5 ± 0.4
C_3A	3E-02 ± 5E-02	7E-03 ± 2E-03	1E-03 ± 1E-03	1.3E-02 ± 1E-03
CA_4	n.d.	n.d.	n.d.	n.d.
C_2A_3	n.d.	n.d.	n.d.	n.d.
A_2C_3	5E-02 ± 9E-02	9E-03 ± 8E-03	3.3E-03 ± 1E-04	8E-03 ± 4E-03
AC_4	4E-02 ± 5E-02	1E-02 ± 8E-03	1E-02 ± 8E-03	4E-02 ± 3E-02
CA_5	n.d.	n.d.	n.d.	n.d.
C_2A_4	n.d.	n.d.	n.d.	n.d.
A_3C_3	8E-04 ± 1E-04	n.d.	n.d.	n.d.
A_2C_4	4E-04 ± 2E-04	1.3E-03 ± 2E-04	6E-04 ± 5E-04	4E-04 ± 3E-04
A_2C_4	2E-04 ± 3E-04	6E-04 ± 9E-04	n.d.	n.d.

S3. Principal Component Analysis.

The spectrum of the eigenvalues and the eigenvector compositions, as obtained by the diagonalization of **C** as described in the main text are reported in the Table S2

Table S2. Results from Principal Component Analysis

**RR**	**RS**	**SR**	**SS**
Eigenvalues	Eigenvalues	Eigenvalues	Eigenvalues
1 1.21	1 1.6	1 1.8	6 1.7
2 0.42	2 0.46	2 0.69	5 0.56
3 0.12	3 0.13	3 0.12	4 0.18
4 2.6E-002	4 8.0E-003	4 1.4E-002	3 5.2E-003
5 4.8E-004	5 6.9E-004	5 1.4E-004	2 2.1E-004
6 1.2E-010	6 3.1E-010	6 -1.2E-009	1 2.0E-009
Composition of the eigenvector 1	Composition of the eigenvector 1	Composition of the eigenvector 1	Composition of the eigenvector 1
1 0.66	1 0.69	1 0.75	1 0.69

2 -0.75<br>3 5.9E-002<br>4 2.5E-002<br>5 1.3E-003<br>6 -6.8E-005<br>--------------<br>Composition of the eigenvector 2<br>1 0.49<br>2 0.37<br>3 -0.79<br>4 -5.0E-002<br>5 -1.2E-002<br>6 -2.1E-004	2 -0.72<br>3 5.7E-002<br>4 -1.9E-002<br>5 -2.7E-003<br>6 -4.7E-005<br>--------------<br>Composition of the eigenvector 2<br>1 0.49<br>2 0.41<br>3 -0.76<br>4 -0.13<br>5 -7.1E-003<br>6 -1.2E-004	2 -0.66<br>3 -6.5E-002<br>4 -2.8E-002<br>5 -2.8E-003<br>6 -4.4E-005<br>--------------<br>Composition of the eigenvector 2<br>1 0.37<br>2 0.50<br>3 -0.76<br>4 -0.10<br>5 -4.9E-003<br>6 -2.3E-005	2 -0.72<br>3 1.9E-002<br>4 1.2E-002<br>5 2.2E-004<br>6 -9.4E-005<br>--------------<br>Composition of the eigenvector 2<br>1 -0.45<br>2 -0.41<br>3 0.79<br>4 5.7E-002<br>5 1.4E-003<br>6 1.2E-004

From the table we can observe: i - in both the diastereoisomeric pairs (RR/RS and SS/SR) the species resulting less soluble in acetonitrile at 25°C (i.e. RS and SR) show a systematically higher ratio of the between the forst an second eigenvalue;

ii - the eigenvector compositions (only the first two eigenvectors were take into account representing between the 88% and 91% of the whole variance) are essentially the same in all the simulations. However the species resulting less soluble in acetonitrile at 25°C (i.e. RS and SR) systematically show a more pronounced contribution of the fourth component (i.e. the tetramers) in the second eigenvector.

S4. ECD spectra: details and computational modelling.

Experimental details.

All the spectra were recorded at the temperature of 25 °C at a concentration of 0.5 mM in $CHCl_3$ using a data Pitch of 1 nm, D.I.T 1 s and 10 scans.

Modelling of ECD spectrum.

In this section we describe the computational strategy utilized for modelling the ECD spectrum of (R)-1,3-diphenyl-3-(phenylamino)propan-1-one (hereafter **1**).

The standard strategy followed for this purpose was based on Time Dependent Density Functional Theory (TD-DFT) calculations carried out on a series of structures extracted from MD simulation.

Step 1. Conformational sampling.

1 was simulated through Molecular Dynamics (MD) simulation in acetonitrile (453 molecules) at the temperature of 25°C and at the pressure of 1.0 bar. The simulation box is pictorially represented in Figure S1. The program Gromacs was used usulizing the same protocol outlined in the main text.

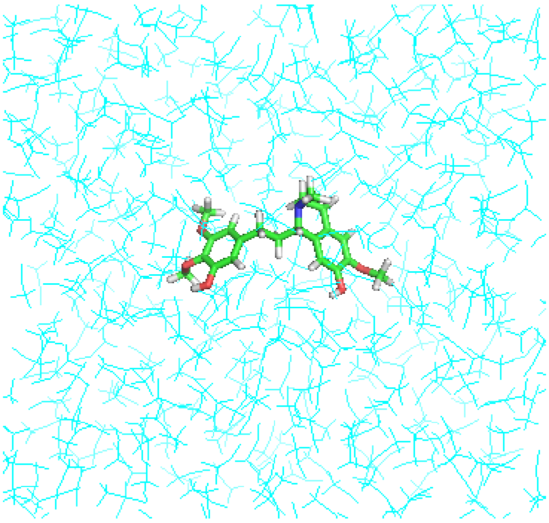

Figure S1. Simulation box of ***1*** *in acetonitrile (blue).*

The simulation was propagated for 60 ns. At the end, the conformational space of **1** was analyzed through Essential Dynamics.[ii]

Briefly: the all-atom covariance matrix of the **1** was constructed, after removing the corresponding roto-translational degrees of freedom, and diagonalized. This operation produces a set of eigenvectors and associated eigenvalues whose spectrum is represented in Figure S2.

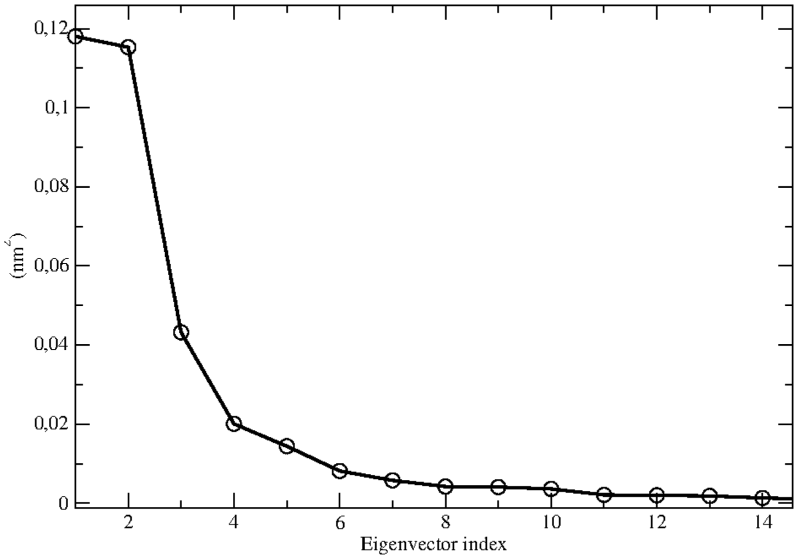

Figure S2. *Spectrum of the eigenvalues of the all-atom covariance matrix of* ***1*** *in acetonitrile (blue) evaluated from a MD simulation of 60 ns..*

As usual the eigenvectors showing higher eigenvalues describe the internal (generalized) coordinate of **1** responsible of the corresponding large amplitude fluctuations. On the other hand the eigenvectors characterized by low eigenvalues describe quasi-harmonic, and hence conformationally irrelevant, eigendirections. The spectrum clearly shows that the first two eigenvalues are much higher than the others accounting for more than 50% of the whole

fluctuations. This means that the conformational repertoire of **1** can be safely and exhaustively determined by using these two eigenvectors ($\mathbf{v}_1$ and $\mathbf{v}_2$).

The projection of the MD simulation, i.e. the sequence of **1** cartesian coordinate onto $\mathbf{v}_1$ and $\mathbf{v}_2$. produces the free-energylansdcape (virtually coincident with probability landscape) reported in Figure S3.

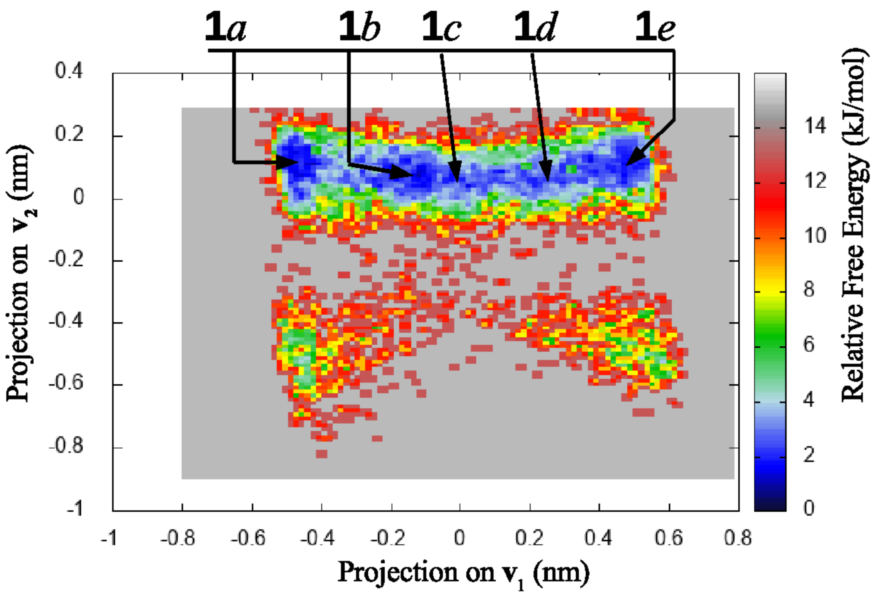

Figure S3. *Free energy landscape of* **1** *in the space formed by the first two eigenvectors of all-atom covariance matrix.*

In This Figure we observe a conformational region (in blue) much more stable than the rest of the conformational space, from which we extracted 5 representative structures (**1***a*-*e*). These structures are reported in Figure S4.

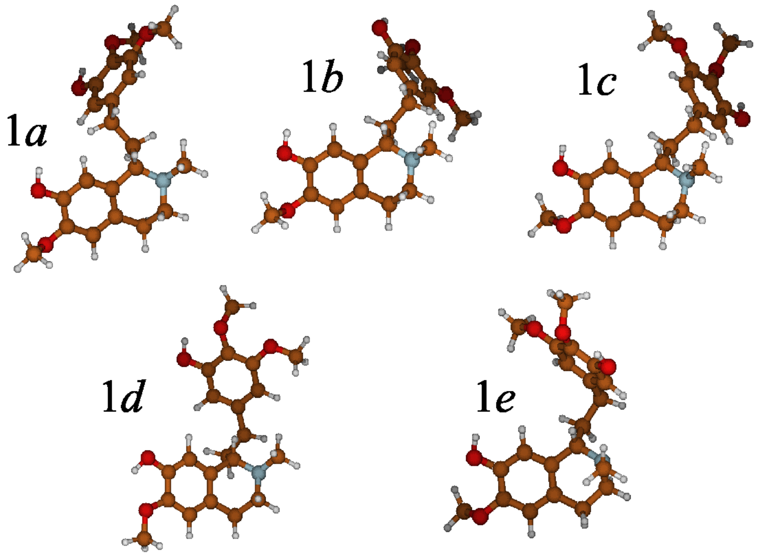

Figure S4. *Schematic view of the 5 strucyìtures representing the most stable sub-region of the conformational space (Fig S3).*

Step 2. Quantum-chemical calculations.

Each of the above five structures were used for calculating the corresponding spectrum of 1 in acetonitrile.

For this purpose we adopted, for each of the five structures, the following protocol.

2a) *Constrained minimization*: the geometry was optimized at the B3LYP/6-311+G** level of theory in the set of quasi-harmonic internal coordinates excluded by the previous conformational analysis. In other words we blocked the torsion angles (proper dihedrals) of **1** relaxing the bond distances, bond angles and improper dihedrals.

The cartesian coordinates of the consttrained oprimized structures are here reported in the Table S3, the corresponding excitation energies are reported in the Table S4.

Table S3. Collection of the cartesian coordinates utilized for the ECD spectra calculation

1a

C	-4.430038	-0.260060	1.116882
C	-3.439722	0.716404	0.966330
C	-2.574856	0.651499	-0.134702
C	-2.749560	-0.377872	-1.070813
C	-3.723690	-1.361715	-0.905397
C	-4.580873	-1.305476	0.209766
C	-1.445870	1.658244	-0.316833
N	-1.624252	2.903762	0.464397
C	-2.928003	3.127778	1.074127

C	-3.337434	1.904059	1.891961
C	-0.059280	1.064967	0.069807
C	0.570644	0.100407	-0.953449
C	1.967078	-0.333311	-0.554892
C	3.077062	0.434308	-0.943811
C	4.369395	0.066351	-0.554132
C	4.550344	-1.072640	0.246930
C	3.442231	-1.842617	0.620098
C	2.153963	-1.477191	0.221856
O	5.790646	-1.546521	0.641235
C	6.505771	-0.709409	1.560537
O	5.510285	0.737576	-0.900053
C	5.385824	1.886342	-1.723230
O	3.632755	-2.962695	1.368494
C	-1.135547	4.071397	-0.246258
O	-3.891153	-2.392550	-1.794582
O	-5.528461	-2.269194	0.456004
C	-6.597678	-2.354703	-0.488905
H	6.788744	0.233810	1.086044
H	5.903741	-0.505834	2.455489
H	7.403218	-1.265927	1.841334
H	4.781579	2.666255	-1.241432
H	6.401535	2.257186	-1.873300
H	4.942886	1.637112	-2.696123
H	2.920969	1.312257	-1.560782
H	1.315421	-2.095560	0.527376
H	4.599928	-3.050424	1.466109
H	-0.060841	-0.787835	-1.064013
H	0.603966	0.591841	-1.936306
H	-0.147205	0.572254	1.045337
H	0.627015	1.907709	0.215658
H	-1.413171	1.909875	-1.393795
H	-1.131380	4.937993	0.424685
H	-0.105795	3.907405	-0.582047
H	-1.743987	4.330868	-1.135931
H	-2.843344	4.011709	1.718727
H	-3.720984	3.345779	0.329665
H	-4.298341	2.083098	2.388001
H	-2.588656	1.740463	2.679634
H	-2.116430	-0.416250	-1.955984
H	-3.216015	-2.314944	-2.486472
H	-5.116561	-0.222101	1.958627
H	-7.260124	-3.142177	-0.121890
H	-7.152705	-1.407722	-0.536144
H	-6.234830	-2.616681	-1.487247

1b

C	-4.218981	0.034216	1.415515
C	-3.217939	0.801828	0.812519
C	-2.648171	0.363527	-0.388555
C	-3.088058	-0.839405	-0.953771
C	-4.069405	-1.615706	-0.338349

C	-4.650858	-1.170100	0.864863
C	-1.503887	1.137716	-1.032213
N	-1.508825	2.594098	-0.727839
C	-2.550726	3.085093	0.172958
C	-2.769754	2.138431	1.351634
C	-0.154087	0.512744	-0.585789
C	1.049757	0.943210	-1.445335
C	2.349889	0.317190	-0.984725
C	3.083583	0.916716	0.052159
C	4.267165	0.333504	0.516162
C	4.711563	-0.872333	-0.051094
C	3.984350	-1.458886	-1.093107
C	2.807602	-0.868428	-1.560604
O	5.893343	-1.501101	0.305219
C	5.926350	-2.042448	1.633115
O	5.057443	0.852326	1.504784
C	4.669345	2.085084	2.090974
O	4.441655	-2.609738	-1.658101
C	-1.432879	3.425817	-1.920865
O	-4.500817	-2.810112	-0.856193
O	-5.588128	-1.911489	1.542874
C	-6.842183	-2.094294	0.881580
H	5.877936	-1.244285	2.378301
H	5.097825	-2.744985	1.790968
H	6.877908	-2.572508	1.720237
H	5.438350	2.318972	2.829643
H	4.626080	2.888386	1.344434
H	3.695595	2.006180	2.591930
H	2.724062	1.844701	0.482565
H	2.268030	-1.347262	-2.372159
H	5.295635	-2.792626	-1.222188
H	0.860392	0.669115	-2.491845
H	1.137147	2.035709	-1.409157
H	-0.237061	-0.581230	-0.626199
H	0.020402	0.779501	0.462812
H	-1.591699	1.006223	-2.121535
H	-2.352244	3.391070	-2.538749
H	-1.268931	4.469934	-1.628362
H	-0.590568	3.120516	-2.548289
H	-2.241114	4.077561	0.526762
H	-3.520574	3.218734	-0.347758
H	-3.521835	2.556518	2.030280
H	-1.834798	2.047319	1.922976
H	-2.648285	-1.189810	-1.888038
H	-3.993563	-2.997118	-1.661361
H	-4.686299	0.361340	2.340654
H	-6.731264	-2.669403	-0.042553
H	-7.473454	-2.645380	1.582676
H	-7.309925	-1.125812	0.657732

1c

C	-4.465753	0.004037	1.156383

C	-3.472291	0.852232	0.656616
C	-2.723962	0.449281	-0.455391
C	-3.001072	-0.786355	-1.053308
C	-3.997174	-1.626337	-0.557974
C	-4.734437	-1.232859	0.576068
C	-1.568325	1.300433	-0.966121
N	-1.695346	2.751142	-0.662739
C	-2.896726	3.172716	0.057563
C	-3.210067	2.228934	1.217212
C	-0.243498	0.751994	-0.371354
C	1.030717	1.380174	-0.966410
C	2.298132	0.780724	-0.391318
C	2.893006	-0.330624	-1.013256
C	4.036220	-0.924276	-0.468491
C	4.604262	-0.387604	0.699006
C	4.001747	0.712172	1.320823
C	2.852674	1.296460	0.779868
O	5.711064	-0.925406	1.334755
C	6.946289	-0.825084	0.612719
O	4.675888	-2.012998	-0.995718
C	4.121189	-2.617804	-2.152551
O	4.544975	1.210157	2.464779
C	-1.484100	3.590302	-1.834827
O	-4.311083	-2.827640	-1.141436
O	-5.766723	-1.990552	1.074229
C	-5.403465	-3.257286	1.628332
H	7.156958	0.216613	0.338087
H	7.723276	-1.189609	1.289145
H	6.919992	-1.443537	-0.288266
H	4.114844	-1.928256	-3.007129
H	4.764005	-3.470053	-2.381319
H	3.098811	-2.972654	-1.968873
H	2.451698	-0.725150	-1.921712
H	2.413073	2.151616	1.284371
H	5.289194	0.617391	2.682232
H	1.023633	1.246013	-2.057266
H	1.014033	2.458398	-0.773311
H	-0.210676	-0.334195	-0.530292
H	-0.258596	0.911092	0.713375
H	-1.523797	1.171759	-2.059647
H	-0.524425	3.358338	-2.305297
H	-2.277951	3.479868	-2.600075
H	-1.459165	4.642956	-1.529492
H	-2.718355	4.190843	0.427324
H	-3.784818	3.220932	-0.604252
H	-4.083609	2.593326	1.769665
H	-2.362740	2.225334	1.917827
H	-2.434851	-1.100182	-1.930875
H	-3.737439	-2.952782	-1.913231
H	-5.067924	0.301146	2.011002
H	-6.319947	-3.672318	2.054569
H	-5.013781	-3.935676	0.863259

H	-4.657205	-3.135064	2.424944

1d

C	-4.594009	0.107647	0.090113
C	-3.442757	0.895289	0.236759
C	-2.185225	0.351668	-0.044237
C	-2.083535	-0.980157	-0.468844
C	-3.220481	-1.766291	-0.607237
C	-4.486109	-1.216523	-0.320576
C	-0.932745	1.184424	0.196828
N	-1.119761	2.642130	-0.042561
C	-2.488717	3.118112	-0.240767
C	-3.497058	2.354660	0.619069
C	-0.431141	0.953273	1.649029
C	1.037218	1.357357	1.884842
C	2.064875	0.479531	1.192704
C	2.998476	1.053711	0.315106
C	3.965834	0.265450	-0.321396
C	4.011740	-1.111748	-0.060269
C	3.074324	-1.683867	0.809967
C	2.105315	-0.896472	1.434167
O	4.899036	-1.983649	-0.668906
C	6.278408	-1.803356	-0.319588
O	4.901015	0.738272	-1.200668
C	4.869515	2.117352	-1.533019
O	3.115188	-3.024030	1.040050
C	-0.245186	3.177505	-1.075006
O	-3.115395	-3.061234	-1.024326
O	-5.527604	-2.099526	-0.502960
C	-6.845744	-1.636201	-0.262493
H	6.821140	-2.622258	-0.797936
H	6.650583	-0.845378	-0.691779
H	6.417525	-1.855382	0.767998
H	3.907310	2.401126	-1.978218
H	5.061485	2.749931	-0.656342
H	5.664536	2.265324	-2.266337
H	2.966103	2.123007	0.140081
H	1.403758	-1.376702	2.108859
H	3.818295	-3.368470	0.457296
H	1.172204	2.401059	1.577710
H	1.225317	1.332796	2.967778
H	-0.558354	-0.105998	1.903613
H	-1.074639	1.523488	2.328470
H	-0.155976	0.820030	-0.486737
H	-0.293823	4.273675	-1.063926
H	0.791740	2.885374	-0.884522
H	-0.512902	2.841871	-2.096874
H	-2.511580	4.191039	-0.004130
H	-2.804787	3.017503	-1.298735
H	-4.500868	2.764799	0.458107
H	-3.258835	2.502901	1.682333
H	-1.117203	-1.424768	-0.691508

H	-4.019261	-3.420312	-1.054910
H	-5.566607	0.543892	0.295818
H	-7.102520	-0.800680	-0.926775
H	-7.507033	-2.479874	-0.468834
H	-6.973190	-1.319739	0.781002

1e

C	4.431664	-0.247530	-0.440445
C	3.326962	-1.060944	-0.148825
C	2.072208	-0.459773	0.023038
C	1.956995	0.930125	-0.129009
C	3.053395	1.728783	-0.439658
C	4.321645	1.130848	-0.583318
C	0.827436	-1.279652	0.378448
N	0.985578	-2.722195	0.120438
C	2.271127	-3.229100	0.605383
C	3.487488	-2.566954	-0.062196
C	0.395082	-1.043060	1.846203
C	-1.078248	-1.401540	2.156334
C	-2.095265	-0.811640	1.200461
C	-2.428196	0.551805	1.290634
C	-3.350877	1.121290	0.406896
C	-3.960842	0.315343	-0.569043
C	-3.620190	-1.038768	-0.661632
C	-2.693079	-1.603653	0.219703
O	-4.852911	0.793151	-1.514161
C	-6.118828	1.232769	-1.001474
O	-3.729693	2.435932	0.413447
C	-3.144506	3.294842	1.378755
O	-4.197892	-1.805938	-1.625089
C	0.723974	-3.081739	-1.271609
O	2.956853	3.084982	-0.627238
O	5.435139	1.855525	-0.936940
C	5.883231	2.817449	0.019693
H	-5.995982	2.119353	-0.374082
H	-6.729551	1.478609	-1.873495
H	-6.605584	0.436634	-0.423651
H	-3.571119	4.283053	1.196990
H	-3.383877	2.977240	2.402020
H	-2.053609	3.343871	1.264749
H	-1.965885	1.157180	2.062593
H	-2.453922	-2.658319	0.128341
H	-4.751776	-1.196305	-2.148829
H	-1.178233	-2.492533	2.143163
H	-1.299201	-1.075623	3.181030
H	0.552494	0.018824	2.076640
H	1.049730	-1.597708	2.527675
H	0.008078	-0.933574	-0.260109
H	0.814037	-4.168231	-1.385723
H	-0.302107	-2.800056	-1.530217
H	1.398692	-2.604805	-2.004807
H	2.317894	-3.063375	1.688289

H	2.293752	-4.314688	0.453023
H	3.625055	-2.976517	-1.073474
H	4.400846	-2.823399	0.490814
H	0.977453	1.398211	-0.020487
H	2.024967	3.338541	-0.539013
H	5.416793	-0.687444	-0.575313
H	5.135646	3.598794	0.187597
H	6.129199	2.333164	0.974758
H	6.788132	3.261853	-0.401751

2a) *Excitation energies and moments*: Time-Dependent Density Functional Theory (TD-DFT) calculations were performed at the same level of theory using the above constrained-optimized structures and in the presence of an implicit solvation model (Polarizable Continuum Model). The transition energies (eV) and moments (expressed as oscillator strength) as well as the corresponding oscillator strenghts are below reported in the Table S4*(a)* and Table S4*(b)*, respectively.

Table S4:
(a) Transition energies and oscillator strengths
1a

4.5417	0.0085
4.7667	0.0577
4.8997	0.0993
4.9305	0.0016
5.0224	0.0471
5.1885	0.0116
5.2457	0.0012
5.2571	0.0211
5.2691	0.0058
5.2757	0.0135
5.3506	0.0109
5.3799	0.0201
5.3960	0.0096
5.4210	0.0004
5.4350	0.0316
5.4780	0.0248
5.4926	0.0261
5.5383	0.0150
5.6359	0.0122
5.6538	0.0086
5.6889	0.0032
5.7067	0.0069
5.7316	0.0256
5.7504	0.0141
5.7732	0.0003
5.7866	0.0112
5.8395	0.0194
5.8774	0.0197
5.8937	0.0330

5.9450	0.1205
5.9547	0.0349
5.9765	0.3332
5.9922	0.0234
5.9932	0.1439
6.0146	0.0402
6.0191	0.3225
6.0752	0.0854
6.0906	0.0522
6.0984	0.0155
6.1301	0.0389
6.1747	0.1407
6.1870	0.0829
6.1917	0.2332
6.2096	0.0408
6.2204	0.1385
6.2337	0.0141
6.2792	0.0026
6.2820	0.0088
6.2958	0.0184
6.3093	0.0034
6.3099	0.0160
6.3464	0.0093
6.3772	0.0047
6.3840	0.0057
6.3951	0.0045
6.3965	0.0314
6.4076	0.0119
6.4369	0.0514
6.4548	0.0098
6.4659	0.0090
6.4732	0.0424
6.4927	0.0299
6.5075	0.0014
6.5218	0.0062
6.5807	0.0038

1b

4.5042	0.0183
4.7600	0.0869
4.8772	0.0755
4.9164	0.0064
5.0998	0.0104
5.1962	0.0233
5.2109	0.0493
5.2644	0.0163
5.2770	0.0022
5.2903	0.0042
5.3376	0.0307
5.3528	0.0058
5.4073	0.0028
5.4212	0.0055

5.4636 0.0113
5.4984 0.0428
5.5269 0.0001
5.5363 0.0190
5.6352 0.0370
5.6677 0.0011
5.6737 0.0307
5.7096 0.0009
5.7240 0.0224
5.7682 0.0037
5.8119 0.0157
5.8337 0.0095
5.8440 0.0241
5.8749 0.0646
5.8993 0.0272
5.9029 0.0142
5.9170 0.0721
5.9454 0.1653
5.9655 0.1113
6.0133 0.2978
6.0326 0.1173
6.0570 0.0251
6.0623 0.0368
6.0700 0.0587
6.0826 0.1532
6.1176 0.0197
6.1305 0.1867
6.1592 0.0396
6.1721 0.0199
6.1905 0.0298
6.2289 0.2303
6.2459 0.0327
6.2739 0.0099
6.2886 0.0187
6.3043 0.0045
6.3080 0.0275
6.3213 0.0189
6.3499 0.0016
6.3594 0.0201
6.3666 0.0260
6.3744 0.0175
6.3836 0.0014
6.4030 0.0052
6.4098 0.0125
6.4241 0.0066
6.4505 0.0111
6.4615 0.1423
6.4742 0.0937
6.5038 0.0100
6.5066 0.0062
6.5543 0.0007

1c

4.5317	0.0223
4.7698	0.0782
4.9031	0.0663
4.9143	0.0200
5.1070	0.0112
5.2022	0.0623
5.2214	0.0064
5.2806	0.0011
5.2957	0.0043
5.3095	0.0177
5.3492	0.0235
5.3907	0.0036
5.4029	0.0005
5.4139	0.0099
5.4651	0.0478
5.5145	0.0019
5.5298	0.0153
5.5317	0.0181
5.6610	0.0422
5.6687	0.0178
5.7138	0.0192
5.7248	0.0246
5.7297	0.0062
5.7586	0.0157
5.7811	0.0359
5.7956	0.0373
5.8400	0.0061
5.8617	0.0105
5.9080	0.0635
5.9205	0.0414
5.9401	0.1548
5.9807	0.0901
5.9977	0.2576
6.0190	0.0364
6.0279	0.0423
6.0455	0.0246
6.0524	0.1393
6.0789	0.0906
6.0910	0.0334
6.1160	0.1452
6.1235	0.0896
6.1741	0.0760
6.2027	0.0288
6.2318	0.0644
6.2334	0.1228
6.2379	0.0442
6.2517	0.1257
6.2670	0.0290
6.2749	0.0090
6.3117	0.0009
6.3213	0.0091

6.3577 0.0252
6.3699 0.0090
6.3747 0.0178
6.3842 0.0055
6.3871 0.0078
6.4057 0.0067
6.4136 0.0289
6.4208 0.0068
6.4314 0.0124
6.4497 0.0084
6.4848 0.0352
6.5014 0.2023
6.5263 0.0034
6.5431 0.0129

1d
4.5990 0.0156
4.7197 0.0980
4.7785 0.0214
4.8942 0.0117
5.0008 0.0090
5.0143 0.0070
5.0389 0.0115
5.1387 0.0093
5.2085 0.0066
5.2517 0.0025
5.2988 0.0534
5.3455 0.0016
5.3695 0.0012
5.3710 0.0197
5.3910 0.0176
5.3958 0.0032
5.4160 0.0110
5.4205 0.0002
5.4415 0.0092
5.4676 0.0652
5.5262 0.0017
5.6094 0.0296
5.6311 0.0032
5.7447 0.0033
5.7612 0.0500
5.7749 0.0126
5.7923 0.1240
5.8191 0.0341
5.8308 0.0118
5.8371 0.0244
5.8563 0.0161
5.8596 0.0090
5.9024 0.0241
5.9102 0.0057
5.9311 0.2560
5.9509 0.0107

5.9814 0.1382
5.9955 0.0795
6.0138 0.3336
6.0286 0.0367
6.0652 0.0282
6.0987 0.0215
6.1093 0.0022
6.1403 0.0074
6.1605 0.0256
6.1872 0.0022
6.2055 0.0259
6.2127 0.0507
6.2383 0.0240
6.2436 0.0152
6.2464 0.0080
6.2678 0.0369
6.2731 0.0103
6.2825 0.0409
6.2977 0.1140
6.3344 0.0535
6.3449 0.0732
6.3530 0.0051
6.3708 0.0630
6.3949 0.2934
6.3979 0.1957
6.4347 0.0060
6.4618 0.0014
6.4681 0.0099
6.4810 0.0526

1e
4.5310 0.0045
4.7500 0.0970
4.8571 0.0067
4.9092 0.0052
5.0427 0.0073
5.1273 0.0028
5.1769 0.0033
5.1847 0.0305
5.2122 0.0506
5.2214 0.0053
5.2518 0.0008
5.2998 0.0088
5.3763 0.0051
5.3868 0.0154
5.4157 0.0250
5.4487 0.0046
5.4864 0.0252
5.5590 0.0070
5.6135 0.0066
5.6182 0.0070
5.6431 0.0041

5.6492	0.0088
5.6895	0.0030
5.7406	0.0843
5.7719	0.0361
5.8254	0.1577
5.8357	0.0215
5.8456	0.0261
5.8493	0.0058
5.8849	0.0385
5.9161	0.2428
5.9382	0.0145
5.9467	0.0282
5.9829	0.0022
5.9962	0.0788
6.0211	0.1085
6.0392	0.1387
6.0748	0.0235
6.0959	0.0995
6.1152	0.0495
6.1569	0.0197
6.1884	0.2148
6.2043	0.1670
6.2289	0.0330
6.2366	0.0230
6.2642	0.0142
6.2778	0.0063
6.2853	0.0480
6.2938	0.0196
6.2954	0.0339
6.3162	0.0477
6.3284	0.0098
6.3403	0.0084
6.3511	0.0213
6.3735	0.0052
6.3935	0.0162
6.4038	0.0013
6.4134	0.0927
6.4280	0.0190
6.4434	0.0390
6.4533	0.1307
6.4766	0.0159
6.4954	0.0102
6.5151	0.0061
6.5179	0.0041

(b) rotatory-strengths in cgs (10^{-40} erg-esu-cm/Gauss) are below reported

1a

1	-2.9184
2	6.4783
3	-24.3235
4	-1.1668
5	3.5535

6	4.8995
7	1.3532
8	1.9252
9	5.5788
10	20.6904
11	-5.6274
12	-14.4562
13	13.1644
14	1.5824
15	-14.6286
16	19.9676
17	-2.9659
18	-12.3390
19	-5.9252
20	23.2810
21	-5.9723
22	8.8980
23	18.1586
24	-10.1124
25	0.4904
26	4.0133
27	8.8503
28	8.4266
29	9.5214
30	-79.6724
31	34.9716
32	-218.7859
33	42.4680
34	144.9930
35	-7.4952
36	-226.1678
37	9.7604
38	-0.9238
39	18.1383
40	20.6243
41	3.8874
42	68.7308
43	19.2954
44	-27.0290
45	50.4879
46	-10.9323
47	-2.2754
48	-2.7564
49	22.5199
50	-0.0722
51	7.8774
52	3.6220
53	-1.3920
54	-7.9987
55	-15.9497
56	-2.8689
57	-1.5846

58	-6.5475
59	6.6355
60	17.7138
61	-2.9506
62	9.7140
63	1.2938
64	-0.8234
65	1.9392

1b

1	-2.3381
2	-2.5271
3	27.1401
4	-7.5810
5	-6.6180
6	3.9135
7	-1.7908
8	20.8847
9	-4.5912
10	1.9699
11	16.9299
12	-7.8252
13	5.2945
14	-3.6139
15	-5.8930
16	-5.1829
17	-1.8397
18	-0.2877
19	0.1303
20	-12.1400
21	-13.7744
22	-6.0774
23	-13.2862
24	0.0795
25	9.8304
26	20.2822
27	-32.6155
28	-30.9712
29	-39.5744
30	-10.7070
31	-17.9896
32	43.2651
33	-128.0509
34	27.9881
35	26.8846
36	13.2575
37	56.0157
38	89.4322
39	-97.2322
40	-53.6128
41	39.5696

42	3.8321
43	-15.1599
44	9.9385
45	-2.1913
46	-15.1035
47	12.5591
48	1.6771
49	-5.8862
50	19.8326
51	30.3395
52	-6.6168
53	-1.0442
54	-4.1668
55	-1.6140
56	5.7935
57	-0.3638
58	9.9487
59	13.9742
60	0.8356
61	-24.6128
62	-30.2925
63	17.9389
64	-5.6646
65	3.8703

1c

1	-2.2498
2	-5.6477
3	-2.6905
4	14.9475
5	-6.7247
6	13.3122
7	-12.9531
8	-1.4633
9	-0.9708
10	16.2499
11	17.1398
12	-5.8323
13	-3.2046
14	-6.4420
15	1.2741
16	3.1554
17	27.5882
18	-1.9672
19	27.4984
20	-19.2451
21	-18.3600
22	32.7341
23	-9.0203
24	2.4021
25	22.8657
26	-2.3610

27	-6.0902
28	6.8018
29	7.4591
30	-5.3027
31	-210.4697
32	-48.4296
33	164.6104
34	-7.8191
35	35.8659
36	24.0846
37	74.8283
38	-256.2160
39	35.3445
40	126.9882
41	33.3308
42	12.8513
43	-16.5029
44	110.3499
45	-149.4327
46	60.2725
47	-44.4975
48	-67.8304
49	1.0786
50	-2.2098
51	-10.1971
52	15.4838
53	-9.4187
54	-23.6824
55	12.8280
56	2.9605
57	-25.1475
58	33.3397
59	-18.5390
60	1.4569
61	19.0699
62	-9.6700
63	63.9623
64	-9.8253
65	17.7335

1d

1	4.8314
2	10.1026
3	-1.0203
4	-9.2717
5	-7.4349
6	8.2684
7	3.6720
8	4.9831
9	3.8851
10	4.0447

11	-10.6338
12	-7.8476
13	3.7887
14	1.4641
15	2.2015
16	5.8421
17	-7.7210
18	-0.3721
19	15.1002
20	-45.3187
21	0.4452
22	-1.2466
23	-2.8624
24	10.6934
25	62.8888
26	-11.8137
27	64.4255
28	3.0433
29	-39.1379
30	41.8793
31	-8.4341
32	9.6171
33	-40.4923
34	10.4639
35	-118.3565
36	-21.7333
37	-16.6501
38	26.8955
39	84.5722
40	7.3171
41	-22.2511
42	21.4443
43	0.9100
44	-4.1106
45	-18.6833
46	-1.5333
47	23.9664
48	59.4572
49	-61.8614
50	-18.8986
51	13.3368
52	-5.5400
53	17.3926
54	-3.9108
55	0.9292
56	-1.4628
57	-11.2474
58	-8.0647
59	15.6454
60	7.4689
61	23.9553
62	-13.4638

63	-3.8453
64	8.6920
65	46.0696

1e

1	-1.4289
2	14.7045
3	0.7986
4	1.8638
5	-5.2804
6	-0.6422
7	0.4931
8	15.0266
9	2.4065
10	-10.4271
11	-1.8168
12	-3.9395
13	-6.9546
14	-6.3293
15	16.5405
16	0.7039
17	10.5400
18	1.8787
19	-11.4702
20	5.8869
21	2.2629
22	18.0523
23	-3.3473
24	-9.7898
25	-2.9784
26	-4.3691
27	-1.5362
28	-36.2599
29	7.7684
30	14.9449
31	-104.6756
32	98.2420
33	44.7120
34	-13.3126
35	65.1909
36	40.1145
37	32.0482
38	15.3306
39	-56.7349
40	-5.2805
41	-1.4191
42	50.7867
43	-101.1891
44	24.9020
45	-19.4684
46	-1.4071
47	-29.2080

48	-49.0934
49	4.4134
50	2.5118
51	-3.3611
52	5.6275
53	-19.1994
54	0.4219
55	-6.8224
56	-17.2643
57	0.6302
58	39.2472
59	5.3683
60	12.3357
61	29.3331
62	-35.3584
63	-1.2208
64	2.2108
65	-15.0862

All the calculations were carried out using Gaussian 16 [iii] (Cineca - Italy is acknowledged for the use of this program).

i Scettri A, Massa A, Palombi L, Villano R, Acocella MR,Organocatalytic asymmetric aza-Michael addition of aniline to chalcones under solvent-free conditions, *Tetrahedron: Asymmetry*, 19, **2008**, 2149-2152.

ii (a) A Amadei , A B Linssen, H J Berendsen Proteins. 1993 , 412-25 (b) I. Daidone, A. Amadei Essential dynamics: foundation and applications Wire (2012) p. 762-770

iii Gaussian 16, Revision C.01,
M. J. Frisch, G. W. Trucks, H. B. Schlegel, G. E. Scuseria,
M. A. Robb, J. R. Cheeseman, G. Scalmani, V. Barone,
G. A. Petersson, H. Nakatsuji, X. Li, M. Caricato, A. V. Marenich,
J. Bloino, B. G. Janesko, R. Gomperts, B. Mennucci, H. P. Hratchian,
J. V. Ortiz, A. F. Izmaylov, J. L. Sonnenberg, D. Williams-Young,
F. Ding, F. Lipparini, F. Egidi, J. Goings, B. Peng, A. Petrone,
T. Henderson, D. Ranasinghe, V. G. Zakrzewski, J. Gao, N. Rega,
G. Zheng, W. Liang, M. Hada, M. Ehara, K. Toyota, R. Fukuda,
J. Hasegawa, M. Ishida, T. Nakajima, Y. Honda, O. Kitao, H. Nakai,
T. Vreven, K. Throssell, J. A. Montgomery, Jr., J. E. Peralta,
F. Ogliaro, M. J. Bearpark, J. J. Heyd, E. N. Brothers, K. N. Kudin,
V. N. Staroverov, T. A. Keith, R. Kobayashi, J. Normand,
K. Raghavachari, A. P. Rendell, J. C. Burant, S. S. Iyengar,
J. Tomasi, M. Cossi, J. M. Millam, M. Klene, C. Adamo, R. Cammi,
J. W. Ochterski, R. L. Martin, K. Morokuma, O. Farkas,
J. B. Foresman, and D. J. Fox, Gaussian, Inc., Wallingford CT, 2019.
